# Tropomyosin-receptor kinase fused gene (TFG) regulates lipid production in human sebocytes

**DOI:** 10.1038/s41598-019-43209-3

**Published:** 2019-04-29

**Authors:** So-Ra Choi, Yul-Lye Hwang, Soo Jung Kim, Kyung-Cheol Sohn, Chong Won Choi, Kyung Duck Park, Young Lee, Young-Joon Seo, Jeung-Hoon Lee, Seung-Phil Hong, Seong Jun Seo, Seong-Jin Kim, Chang Deok Kim

**Affiliations:** 1 Department of Medical Science, School of Medicine, Chungnam National University, Daejeon, Korea; 2 Department of Dermatology, School of Medicine, Chungnam National University, Daejeon, Korea; 3 Department of Dermatology, Dankook University College of Medicine, Cheonan, Korea; 4 Department of Dermatology, Chung-Ang University College of Medicine, Seoul, Korea; 5 Department of Dermatology, Chonnam National University Medical School, Gwangju, Korea

**Keywords:** Waxes, Waxes, Endoplasmic reticulum, Endoplasmic reticulum

## Abstract

The endoplasmic reticulum (ER) is an organelle in which important cellular events such as protein synthesis and lipid production occur. Although many lipid molecules are produced in the ER, the effect of ER-organizing proteins on lipid synthesis in sebocytes has not been completely elucidated. Tropomyosin-receptor kinase fused gene (TFG) is located in ER exit sites and participates in COPII-coated vesicle formation along with many scaffold proteins, such as Sec. 13 and Sec. 16. In this study, we investigated the putative role of TFG in lipid production in sebocytes using an immortalized human sebocyte line. During IGF-1-induced lipogenesis, the level of the TFG protein was increased in a time- and dose-dependent manner. When TFG was over-expressed using recombinant adenovirus, lipid production in sebocytes was increased along with an up-regulation of the expression of lipogenic regulators, such as PPAR-γ, SREBP-1 and SCD. Conversely, down-regulation of TFG using a microRNA (miR) decreased lipid production and the expression of lipogenic regulators. Based on these data, TFG is a novel regulator of lipid synthesis in sebocytes.

## Introduction

Sebocytes are the main cells in the sebaceous glands that produce sebum. Sebum is secreted in a holocrine manner from completely differentiated sebocytes under the control of various hormones^1^. The major components of human sebum are non-polar lipid molecules, including triglycerides, free fatty acids, wax esters, squalene, and cholesterol^2^. Sebum functions as a skin barrier to reduce water loss and prevent the invasion of harmful substances and bacteria from the external environment^3^. In adolescence, sebum production often increases and contributes to establishment of environment favorable to acne induction, such as the colonization of the commensal bacteria *Propionibacterium acnes*^4^.

The endoplasmic reticulum (ER) is the largest intracellular organelle that forms an interconnected network structure. The best-known functions of the ER are to support protein synthesis and folding. Additionally, the ER represents the site of production of lipids such as cholesterol, triacylglycerol and phospholipids^5^. Many enzymes involved in lipid production are located in the ER membrane. For example, sterol response element binding proteins (SREBPs), the pivotal transcription factors involved in lipid synthesis, are present in the ER membrane in an inactive form. When cells are in sterol-deficient state, SREBPs are proteolytically cleaved and released from the ER membrane, subsequently translocating to the nucleus and functioning as transcription factors to induce lipid synthesis^6^. SREBPs regulate the expression of lipogenic transcription factors, including peroxisome proliferator-activated receptor-γ (PPAR-γ), and lipogenic enzymes such as stearoyl-CoA desaturase (SCD) and squalene synthase (farnesyl-diphosphate farnesyltransferase 1, FDFT1), thereby affecting lipid production. The newly synthesized lipids in the ER are exported to other organelles through vesicular and non-vesicular transport systems. In the vesicular transport system, COPII-coated vesicle formation is responsible for transport from the ER to the Golgi apparatus. COPII-coated vesicles contain many scaffolding proteins, including Sec. 13, Sec. 16, Sec. 23, Sar1, and TANGO1. Despite the in-depth understanding of the COPII-coated vesicle transport system, studies are currently underway to delineate the detailed mechanism^7,8^.

Recently, the tropomyosin-receptor kinase fused gene (TFG) was reported to be a novel ER-organizing protein that exerts an important effect on the COPII-coated vesicle transport system. TFG is located in ER exit sites (ERES) and forms hexamers that facilitate the co-assembly of Sec. 16 with COPII subunits. TFG depletion decreases protein export from the ER and induces the accumulation of COPII-coated vesicles throughout the cytoplasm^9,10^. Although the ER is well recognized as the organelle responsible for lipid synthesis and transport, little evidence supporting the effects of ER-organizing proteins on lipid synthesis in sebocytes is available. Since TFG modulates ER function with respect to protein synthesis and transport, we speculate that TFG may also affect lipid synthesis in sebocytes. As shown in this study, TFG regulates lipid production in sebocytes.

## Results

### IGF-1 induces lipid production in immortalized sebocytes

**Figure 1:**
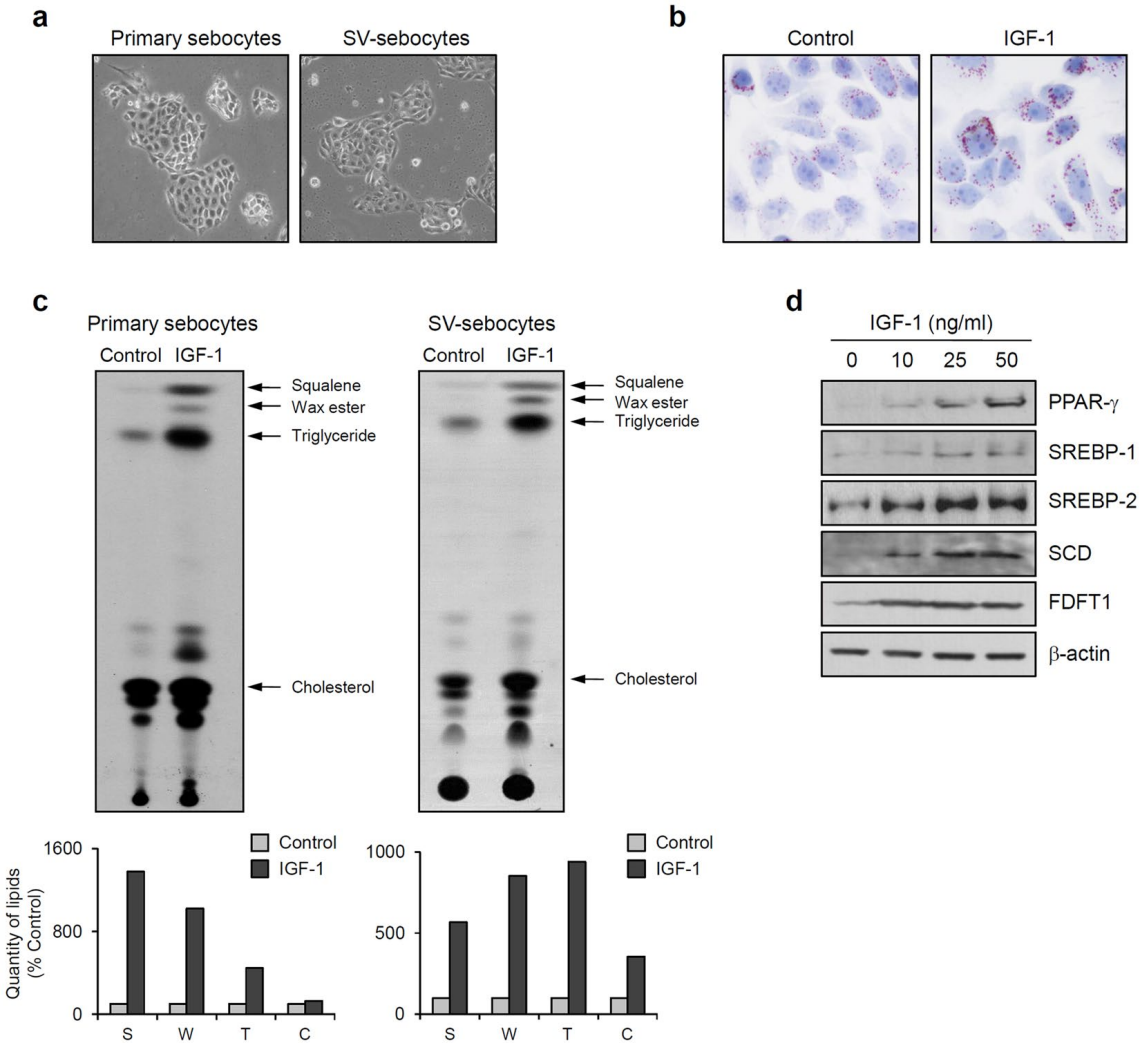
IGF-1 induces lipid production in immortalized sebocytes. (**a**) Primary sebocytes were cultured from isolated sebaceous gland and then immortalized using the recombinant retrovirus expressing simian virus 40 T antigen (SV-sebocytes). (**b**) SV-sebocytes were grown on cover glasses to 70–80% confluence, and then cultured with fresh medium without FBS and rhEGF. After an overnight incubation, cells were treated with IGF-1 (50 ng/ml) for 24 h. Intracellular lipid droplets were visualized using Oil red O staining. IGF-1 increased lipid production in sebocytes. (**c**) Intracellular lipids were analyzed using thin layer chromatography (TLC). IGF-1 increased the production of lipids, including squalene, wax ester, triglyeride and cholesterol. Quantification of lipids was carried out using ImageJ program. Data are represented as a percentage of the control. (**d**) The levels of lipogenic regulators were detected using Western blotting. IGF-1 increased the levels of lipogenic regulatory proteins in a dose-dependent manner. β-Actin was used as a loading control.

We isolated sebaceous glands from skin specimens and established primary sebocyte cultures^11,12^. For the long-term and continuous maintenance of the sebocyte line, we immortalized the primary cultured cells with a recombinant retrovirus expressing simian virus 40 T antigen (SV40T). The morphology of SV40T-transformed sebocytes (SV-sebocytes) resembled primary sebocytes (Fig. 1a). As an IGF-1-induced lipogenesis model was well-established for studies of sebocytes^12,13^, we first assessed whether an IGF-1 treatment induced lipogenesis in our SV-sebocytes. When intracellular lipid droplets were stained with an Oil Red O solution, IGF-1 noticeably increased lipid accumulation in the cytoplasm of SV-sebocytes (Fig. 1b). We analyzed lipid components using thin layer chromatography (TLC), and results clearly showed that IGF-1 significantly increased the production of lipids such as squalene, wax ester, triglyceride, and cholesterol in both the primary sebocytes and SV-sebocytes (Fig. 1c). Various transcription factors, including PPAR-γ, SREBP-1 and SREBP-2, participate in lipid production by up-regulating the expression of lipogenic enzymes, such as SCD and FDFT1. Thus, we next examined the effect of IGF-1 on the expression of those lipogenic regulators. Consistent with the data on lipid production, IGF-1 markedly increased the levels of lipogenic regulatory proteins in a dose-dependent manner (Fig. 1d).

### IGF-1 increases TFG levels in sebocytes

**Figure 2:**
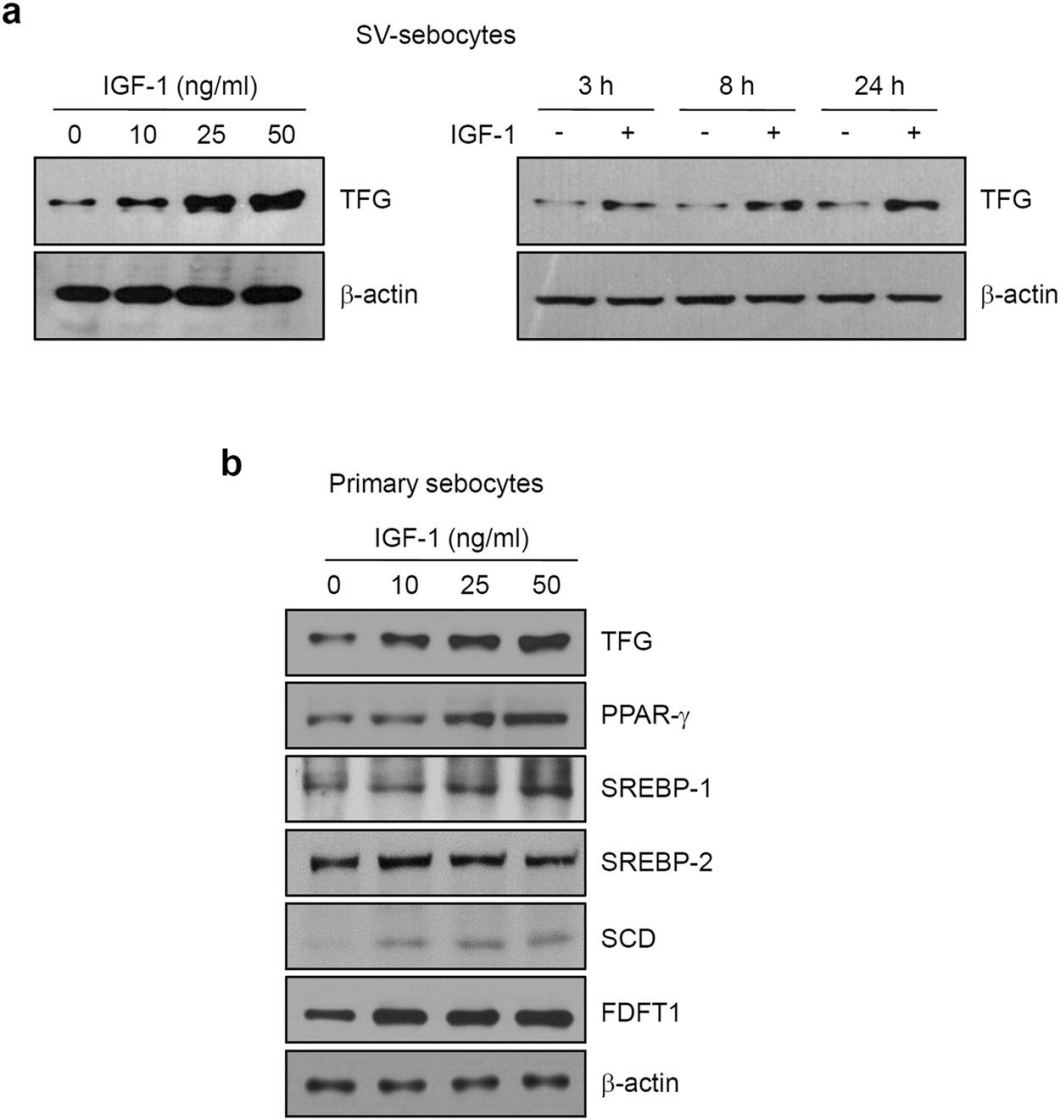
IGF-1 increases TFG levels. (**a**) SV-sebocytes were grown to 70–80% confluence and then received fresh medium without FBS and rhEGF. After an overnight incubation, cells were treated with the indicated concentrations of IGF-1 for 24 h. IGF-1 increased TFG levels in a dose-dependent manner (left panel). In a time-dependent experiment, SV-sebocytes were treated with 50 ng/ml of IGF-1 for the indicated times. IGF-1 increased TFG levels in a time-dependent manner (right panel). (**b**) Primary human sebocytes were treated with the indicated concentrations of IGF-1 for 24 h. IGF-1 increased the levels of TFG and lipogenic regulators in a dose-dependent manner.

TFG is localized in the ERES and participates in the mechanism regulating ER functions^9,10^. Since many lipid molecules are synthesized in the ER, we hypothesized that TFG may have a role in regulating lipid production in sebocytes. We treated SV-sebocytes with IGF-1 and then examined levels of the TFG protein using Western blotting to investigate the possible involvement of TFG in lipid production. IGF-1 increased TFG levels in a dose- and time-dependent manner (Fig. 2a). We repeated this experiment using primary cultured human sebocytes to investigate whether IGF-1 increases TFG levels in non-transformed cells. Consistent with the data obtained from SV-sebocytes, IGF-1 also increased TFG levels in a dose-dependent manner, together with the levels of several lipogenic regulators (Fig. 2b). Based on these results, TFG is involved in the mechanism regulating lipogenesis in sebocytes.

We then investigated which intracellular signaling pathways were important for IGF-1-induced TFG up-regulation. When SV-sebocytes were treated with a PI3K inhibitor (LY294002) or p38 MAPK inhibitor (SB203580), the IGF-1-induced increase in TFG levels was significantly inhibited. Meanwhile, ERK1/2 inhibitor (PD980509) did not block the IGF-1-induced increase in TFG levels. Thus, the PI3K and p38 MAPK signaling pathways are important for IGF-1-induced TFG up-regulation (Supplementary Fig. S1).

### TFG regulates lipid production in sebocytes

**Figure 3:**
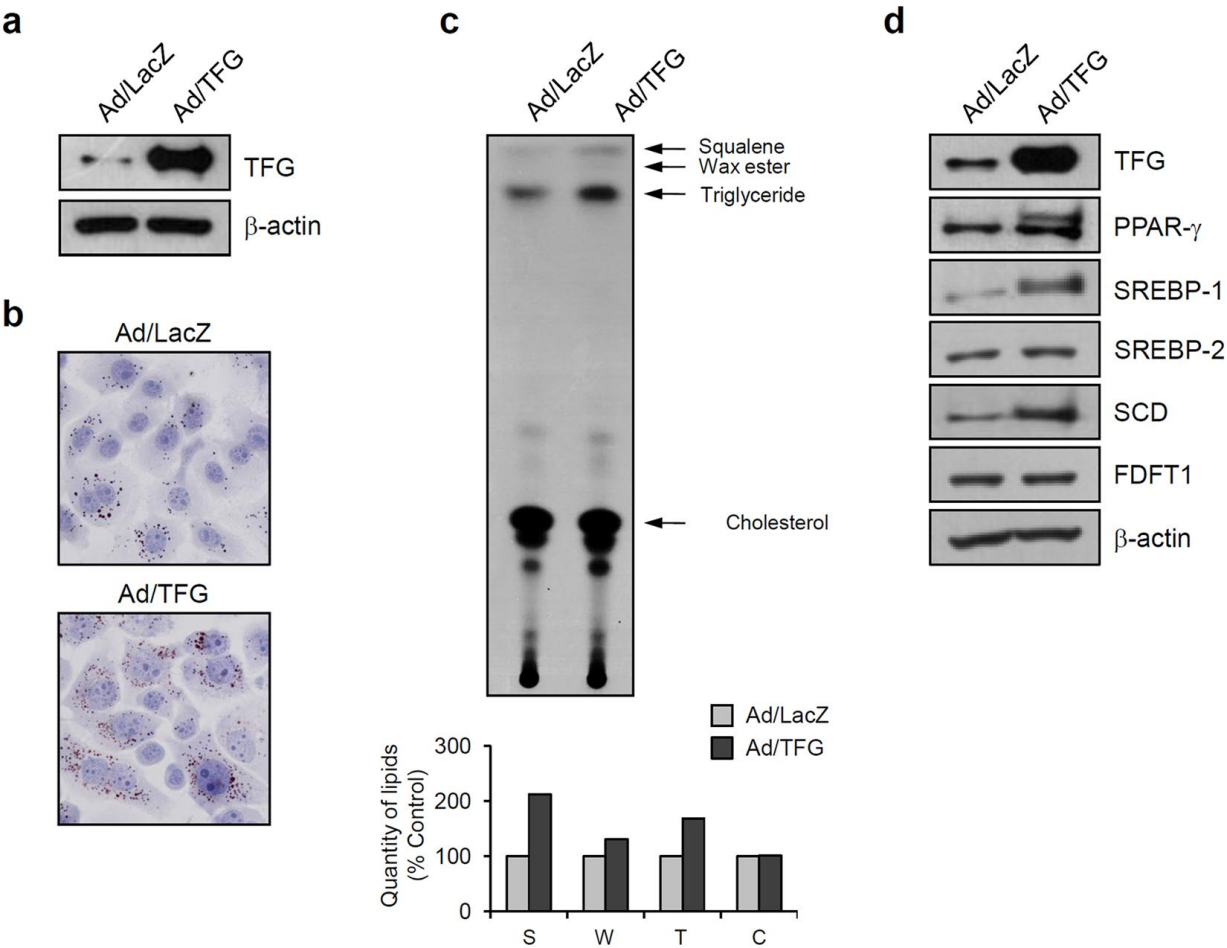
Over-expression of TFG increases lipid production. (**a**) SV-sebocytes were transduced with the recombinant adenovirus expressing TFG (Ad/TFG). Western blot showing increased levels of TFG in Ad/TFG-treated cells compared with the control adenovirus (Ad/LacZ)-treated group. (**b**) Intracellular lipid droplets were detected using Oil red O staining. Over-expression of TFG increased lipid production in sebocytes. (**c**) Intracellular lipids were analyzed using TLC. Over-expression of TFG increased the production of lipids, including squalene and triglyeride. (**d**) Over-expression of TFG increased the levels of lipogenic regulatory proteins, such as PPAR-γ, SREBP-1 and SCD.

We created a recombinant adenovirus expressing TFG to investigate the effect of TFG on lipid production in sebocytes. After transduction with the adenovirus, TFG was expressed at high levels in SV-sebocytes compared with the control adenovirus-treated group (Fig. 3a). Oil Red O staining showed a significant increase in the number of intracellular lipid droplets in cells over-expressing TFG (Fig. 3b). According to the results of the TLC analysis, the levels of specific lipids, including squalene and triglyceride, were increased by TFG over-expression, whereas wax ester and cholesterol levels were not significantly altered (Fig. 3c). We then assessed whether TFG over-expression affected the levels of lipogenic regulators. Over-expression of TFG resulted in a marked increase in the levels of several lipogenic regulators, such as PPAR-γ, SREBP-1 and SCD. However, the levels of the SREBP-2 and FDFT1 proteins were not significantly altered by TFG over-expression (Fig. 3d). When TFG was over-expressed in primary cultured human sebocytes, squalene production and the levels of lipogenic regulators were increased similarly to SV-sebocytes (Supplementary Fig. S2).

**Figure 4:**
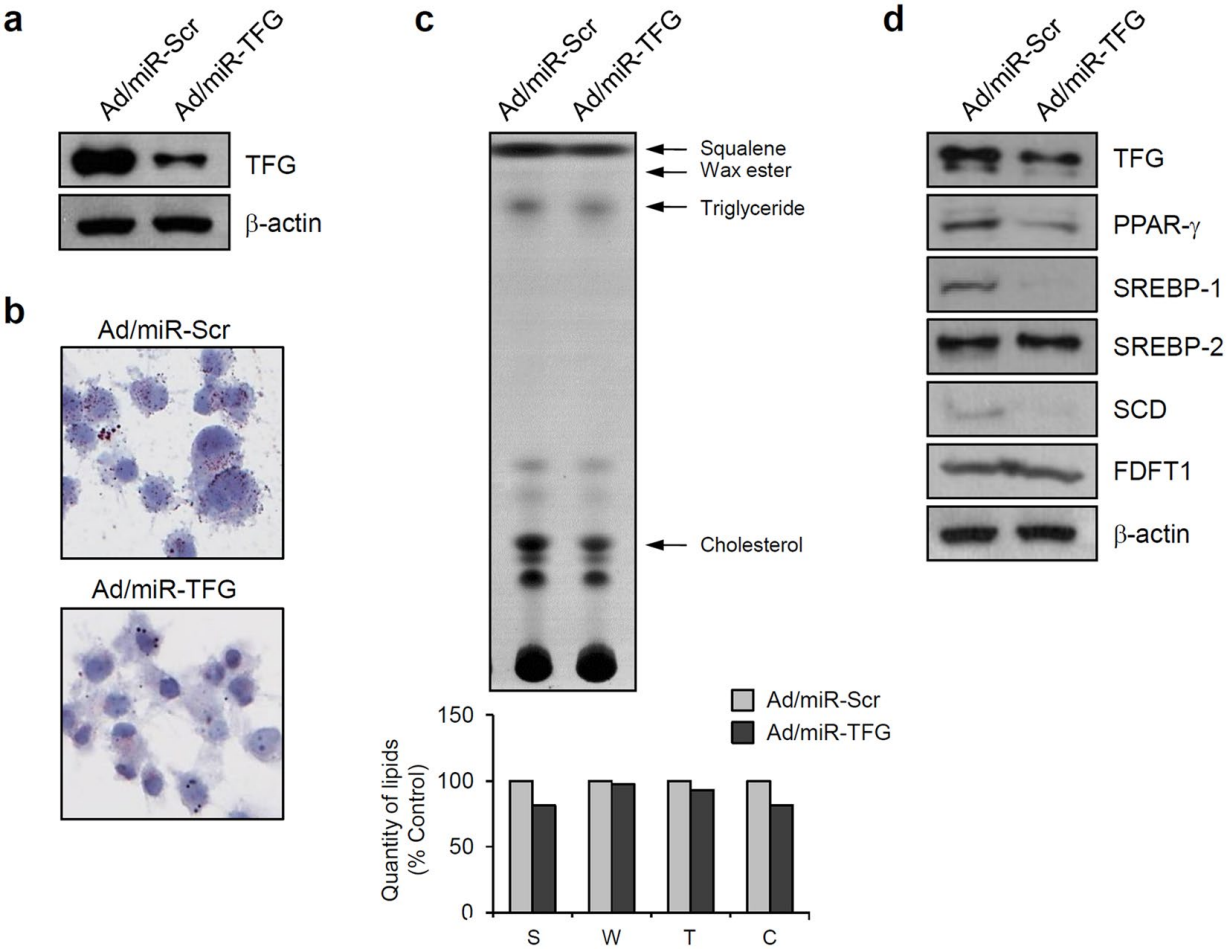
Down-regulation of TFG decreases lipid production. (**a**) SV-sebocytes were transduced with the recombinant adenovirus expressing a microRNA targeting TFG (Ad/miR-TFG). Western blot showing a marked decrease in TFG levels in Ad/miR-TFG-treated cells compared with cells transduced with a control adenovirus expressing a scrambled microRNA (Ad/miR-Scr). (**b**) Intracellular lipid droplets were detected using Oil red O staining. Down-regulation of TFG decreased lipid production in sebocytes. (**c**) Intracellular lipids were analyzed using TLC. Down-regulation of TFG decreased the production of lipids, including squalene, triglyceride, and cholesterol. (**d**) Down-regulation of TFG decreased the levels of lipogenic regulatory proteins, such as PPAR-γ, SREBP-1 and SCD.

Next, we used the recombinant adenovirus expressing a microRNA (miR) targeting TFG (Ad/miR-TFG) to down-regulate its expression. After the transduction of Ad/miR-TFG, the level of the TFG protein was markedly decreased compared to cells transduced with the control adenovirus (Ad/miR-Scr) (Fig. 4a, Supplementary Fig. S3). In contrast to TFG over-expression, down-regulation of TFG decreased the number of intracellular lipid droplets (Fig. 4b). In the TLC analysis, down-regulation of TFG decreased the levels of several lipids, such as squalene, triglyceride and cholesterol (Fig. 4c). In addition, miR-mediated down-regulation of TFG decreased the levels of several lipogenic regulators, including PPAR-γ, SREBP-1 and SCD. Meanwhile, SREBP-2 and FDFT1 levels were not significantly altered by TFG down-regulation (Fig. 4d). Again, the effects of TFG down-regulation were reproducible in primary cultured human sebocytes (Supplementary Fig. S4). Based on these results, TFG regulates lipogenesis in sebocytes by controlling the levels of lipogenic regulators.

Finally, we investigated whether IGF-1-induced lipogenesis was affected by TFG down-regulation. SV-sebocytes were transduced with Ad/miR-TFG and then treated with IGF-1. According to the results of the TLC analysis, IGF-1-induced lipogenesis was slightly inhibited in Ad/miR-TFG-treated cells compared to Ad/miR-Scr-treated cells (Supplementary Figure S5), potentiating the effect of TFG on lipogenesis in sebocytes.

## Discussion

Sebum production is an intrinsic event that occurs in sebocytes. It is closely related to the onset of acne, particularly during adolescence. In this period, excess sebum is produced under the control of many hormones, including androgens, insulin, and IGF-1^1^. Experimentally, IGF-1 induces lipogenesis by inducing the expression of lipogenic regulators, such as PPAR-γ and SREBP-1, in cultured sebocytes^13^. It has been reported that PPAR-γ is activated in the well-fed state and regulates the synthesis of fatty acids and related lipids^14^. SREBP-1 regulates the expression of various genes, whose products mediate the synthesis of cholesterol, fatty acids, and triglycerides^15^. Although some lipogenic signaling pathways and regulatory molecules have been identified, they are not sufficient to understand the complex regulatory mechanism underlying lipid production in sebocytes. In this study, we identified TFG as a novel regulator of lipid production using the IGF-1-induced lipogenesis model of sebocytes. IGF-1 increased TFG levels in sebocytes, and over-expression of TFG increased lipid production, while down-regulation of TFG decreased lipid production.

TFG was initially identified as an oncoprotein that binds to neurotrophic tyrosine kinase receptor type 1 (NTRK1) in thyroid cancer^16^. However, recent reports indicate that TFG is involved in various ER functions, such as COPII-coated vesicle transport, organization of the ER structure, and ER stress^9^. The TFG protein comprises three specific domains, including a Phox and Bem 1p (PB1) domain, a coiled-coil (CC) domain, and a proline and glutamate (P/Q)-rich domain. The P/Q-rich domain of the TFG protein is thought to be important for the ER to Golgi transport^17^. When TFG is depleted, transitional ER elements are dispersed and the export of large proteins, such as procollagen, is defective^18^. Similarly, knock-down of TFG in dermal fibroblasts reduces the levels of collagen 1α1 and collagen 1α2^19^. In pancreatic β-cell specific TFG knockout mice, ER dilation and marked glucose intolerance with reduced insulin secretion are observed^20^. Based on these results, TFG plays an important role in organizing the ER structure, thereby contributing to the synthesis of bio-molecules such as proteins.

We hypothesized that TFG may modulate lipid production in sebocytes, because many lipid molecules are synthesized in the smooth ER. Using recombinant adenoviruses, we showed that TFG actually regulated lipid production in sebocytes. In our experiments, TFG over-expression increased lipogenesis and up-regulated the expression of several lipogenic regulators, such as PPAR-γ, SREBP-1 and SCD. Conversely, down-regulation of TFG decreased lipogenesis and the levels of lipogenic regulators. However, the levels of another lipogenic transcription factor, SREBP-2, and lipogenic enzyme FDFT1 were not significantly altered by TFG over-expression and/or down-regulation. As for Western blot of PPAR-γ, two bands were detected when TFG was over-expressed (Fig. 3d). It has been reported that posttranslational modifications of PPAR-γ are occurred by a variety of stimuli such as EGF, PDGF, TGF-β and insulin, and the posttranslational modifications can affect the activity of PPAR-γ^21^. Elucidation of possibility that posttranslational modification of PPAR-γ is regulated by TFG will be an interesting further study.

Three isoforms of SREBP are expressed in mammalian cells: SREBP-1a, SREBP-1c and SREBP-2. SREBP-1a and SREBP-1c are encoded by a single gene, and each isoform uses a different promoter to produce an alternative amino terminus. Meanwhile, SREBP-2 is encoded by a different gene^22^. The SREBP-1 and SREBP-2 proteins are bound to the ER membrane as the inactive forms until they are activated by proteolytic cleavage. These proteins are similarly transported by COPII-coated vesicles from the ER to Golgi for cleavage and activation^23^. However, SREBP-1 and SREBP-2 can be regulated by different mechanism. For example, insulin enhances the cleavage of SREBP-1c, but not SREBP-2, in rat hepatocytes^24^. Thus, we have speculate that TFG differentially regulates the SREBP-1 and SREBP-2, and the lipid production pathway regulated by TFG is likely related to the transcription factor SREBP-1 in sebocytes. The precise mechanisms by which TFG regulates lipogenic transcription factors will be an interesting topic for further studies.

In summary, TFG regulates lipid production in sebocytes. Our results provide important clues on which to base further investigations of the molecular mechanisms underlying sebum production.

## Materials and Methods

### Cell culture

Human scalp skin tissues were obtained from donors who provided written informed consent. All procedures were approved by the Institutional Review Board of Chungnam National University Hospital. The study was conducted in accordance with the Principles of the Declaration of Helsinki. Skin tissues were sterilized with 70% ethanol for 1 min, and then sebaceous glands were dissected under a stereomicroscope. Isolated sebaceous glands were attached to the culture dish and incubated with Sebomed^®^ medium (Biochrom, Berlin, Germany) supplemented with 10% fetal bovine serum (FBS) and 5 ng/ml recombinant human epidermal growth factor (rhEGF) (Life Technologies Corporation, Grand Island, NY). Generally, sebocytes outgrew from explanted sebaceous gland tissues in 1 week.

For immortalization, the retroviral vector pLXIN-SV40T was transfected into PT67 cells (Clontech Laboratories, Mountain View, CA), a recombinant retrovirus packaging cell line. The medium containing retrovirus was collected, filtered through a 0.22-μm low protein binding filter (Merck KGaA, Darmstadt, Germany) and then transferred to primary cultures of sebocytes. After an overnight infection, the medium containing the retrovirus was replaced with fresh medium and the cells were incubated for two additional days. SV40-transformed cells were selected in medium containing 200 µg/ml G418 (Sigma, St. Louis, MO) for 4 weeks.

### Lipogenesis assay

We employed a well-established IGF-1-induced lipogenesis model^12,13^. Cells were grown to 70–80% confluence and then received fresh medium without FBS and rhEGF. After an overnight incubation, cells were treated with IGF-1 (Sigma). For measurements of lipogenesis, cells were incubated with medium containing 2 μCi of [1-^14^C]acetic acid sodium salt (PerkinElmer, Boston, MA). After two washes with phosphate-buffered saline (PBS), cells were harvested and lipids were extracted with chloroform and methanol (2:1). The solvents were evaporated in a fume hood overnight, and lipids were reconstituted in chloroform. Lipids were then separated using thin layer chromatography (TLC silica gel 60 F_254_, Merck KGaA) with developing buffer consisting of hexane and ethyl acetate (6:1). Lipids were visualized using autoradiography.

### Oil Red O staining

Cells were grown on cover glasses. After treatment, cells were fixed with 10% formaldehyde at room temperature for 1 h, washed with distilled water, and incubated with a 0.7% Oil Red O solution (Sigma) for 0.5–1 h. Cells were then washed vigorously with distilled water and counterstained with hematoxylin.

### Western blot

Cells were lysed in Proprep solution (Intron, Deajeon, Korea). Total protein concentrations were measured using a BCA protein assay kit (Thermo Scientific, Rockford, IL). Samples were run onto SDS-polyacrylamide gels and transferred onto nitrocellulose membranes (Pall Corporation, Port Washington, NY). After blocking with 5% skim milk, the membranes were incubated with primary antibodies. Blots were then incubated with peroxidase-conjugated secondary antibodies and visualized using enhanced chemiluminescence (Intron). The following primary antibodies were used: TFG (Merck KGaA); PPAR-γ (Cell Signaling, Danvers, MA); FDFT1 and SREBP-2 (Abcam, Cambridge, UK); SREBP-1 and β-actin (Santa Cruz, CA, USA); SCD (Thermo Scientific). The following amounts of proteins were loaded for detection using Western blotting: 30 μg for PPAR-γ, SREBP-1, SREBP-2 and SCD; 10 μg for TFG and β-actin; and 5 μg for FDFT1.

### Production of recombinant adenovirus

The TFG cDNA was obtained by reverse transcription-polymerase chain reaction (RT-PCR). Briefly, total RNA was isolated using the Easy-blue RNA extraction kit (Intron). Two micrograms of total RNA were reverse transcribed with Moloney murine leukemia virus (MMLV) reverse transcriptase (ELPIS Biotech, Daejeon, Korea). An aliquot of the RT mixture was subjected to PCR with the primer set for TFG (5′-GTACGGATCCATGAACGGACAGTTGGATCT and 5′-AATTGCGGCCGCTTATCGATAACCAGGTCCAG). The amplified full-length TFG cDNA was subcloned into the pENT/CMV vector and replication-incompetent adenoviruses were created.

For knockdown experiments, we prepared recombinant adenoviruses expressing a microRNA targeting TFG. The target sequences for TFG were designed using BLOCK-iT™ RNAi Designer (Thermo Scientific). The double-stranded DNA oligonucleotides were synthesized and cloned into the parental vector pcDNA6.2-GW/EmGFP-miR (Thermo Scientific). The expression cassette for microRNA was inserted into the pENT/CMV vector, and then the adenovirus was prepared using the method described above. The microRNA sequences were: top strand 5′-TGCTGTTAGCTTCCCACTTAGATCCAGTTTTGGCCACTGACTGACTGGATCTATGGGAAGCTAA, bottom strand 5′-CCTGTTAGCTTCCCATAGATCCAGTCAGTCAGTGGCCAAAACTGGATCTAAGTGGGAAGCTAAC. For adenoviral transduction, sebocytes were incubated with the adenovirus at a multiplicity of infection (MOI) of 10 overnight. Cells were replenished with fresh medium, and incubated for a further 2 d.

## Supplementary information

Supplemenatry information
