# Supplementary material for: Tropomyosin-receptor kinase fused gene (TFG) regulates lipid production in human sebocytes
Source: Sci Rep. 2019 Apr 29;9:6587. doi: 10.1038/s41598-019-43209-3 (PMC6488642; doi:10.1038/s41598-019-43209-3)
Supplement: Supplementary file 1 — Supplemenatry information [file 41598_2019_43209_MOESM1_ESM.pdf]

Supplementary information

Tropomyosin-receptor kinase fused gene (TFG) regulates lipid production in human sebocytes

So-Ra Choi[1,2], Yul-Lye Hwang[2], Soo-Jung Kim[2], Kyung-Cheol Sohn[2], Chong Won Choi[2], Kyung Duck Park[2], Young Lee[1,2], Young-Joon Seo[1,2], Jeung-Hoon Lee[1,2], Seung-Phil Hong[3], Seong-Jun Seo[4], Seong-Jin Kim[5] & Chang-Deok Kim[1,2]

[1]Department of Medical Science, School of Medicine, Chungnam National University, Daejeon, Korea.

[2]Department of Dermatology, School of Medicine, Chungnam National University, Daejeon, Kore.

[3]Department of Dermatology, Dankook University College of Medicine, Cheonan, Korea.

[4]Department of Dermatology, Chung-Ang University College of Medicine, Seoul, Korea.

[5]Department of Dermatology, Chonnam National University Medical School, Gwangju, Korea

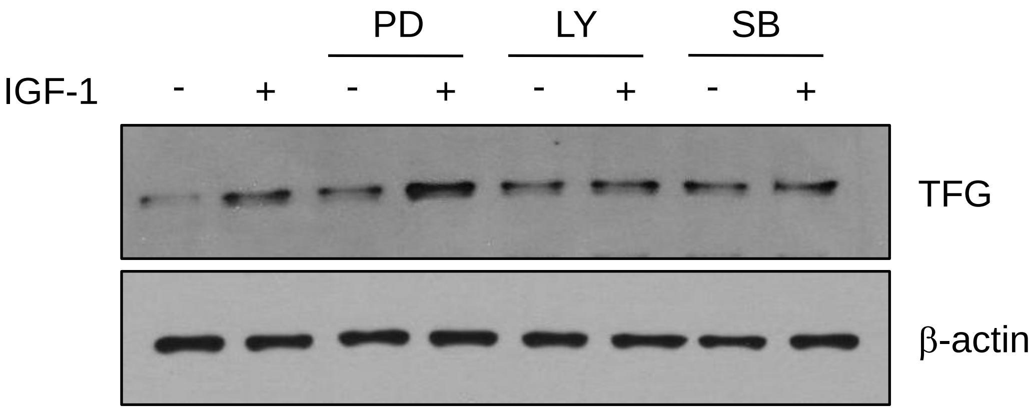

Supplementary Figure S1. SV-sebocytes were grown to 70-80% confluence, then received fresh medium without FBS and rhEGF. After overnight incubation, cells were pretreated with ERK1/2 inhibitor (PD, 25 μM of PD980509), PI3K inhibitor (LY, 20 μM of LY294002) and p38 MAPK inhibitor (SB, 20 μM of SB203580) for 30 min. Cells were then treated with IGF-1 (50 ng/ml) and incubated for 24 h. Inhibition of PI3K and p38 MAPK signaling pathways significantly blocked IGF-1-induced TFG up-regulation.

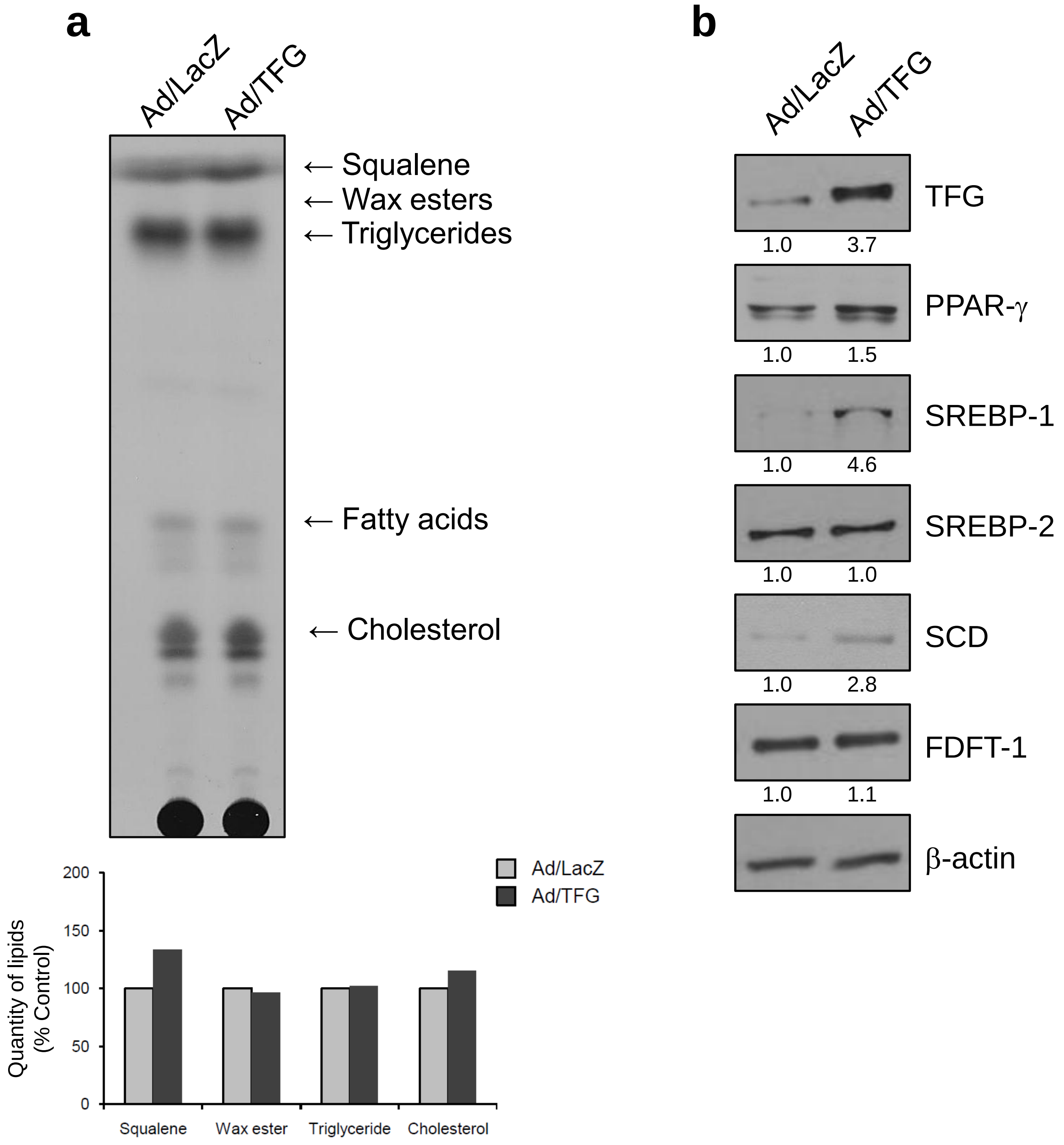

Supplementary Figure S2. (a) Primary cultured human sebocytes were transduced with the recombinant adenovirus expressing TFG (Ad/TFG). Adenovirus expressing LacZ (Ad/LacZ) was used as a negative control. Intracellular lipids were analyzed by TLC. Over-expression of TFG increased the production of lipids including squalene and cholesterol. Quantification of lipids was carried out using ImageJ program. Data are represented as a percentage of the control. (b) Over-expression of TFG increased the protein levels for lipogenic regulators such as SREBP-1 and SCD. The protein levels were normalized to β-actin and represented as fold induction.

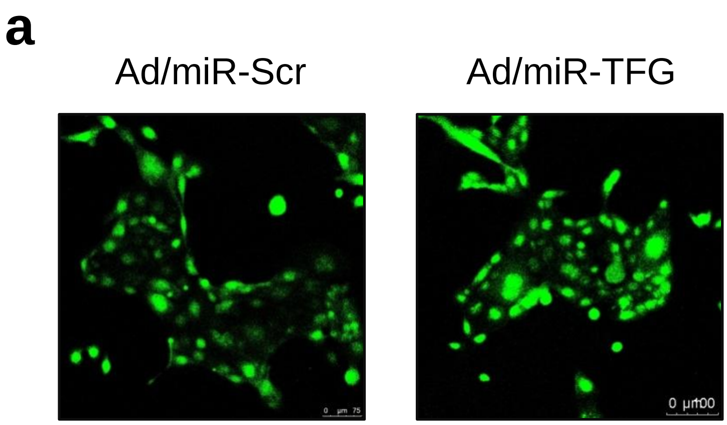

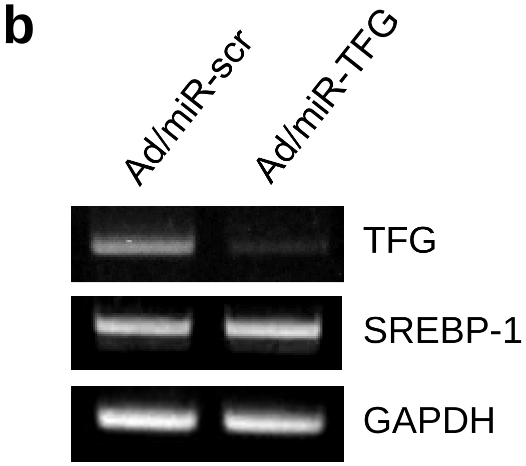

Supplementary Figure S3. (a) SV-sebocytes were transduced with the recombinant adenovirus expressing microRNA targeting TFG (Ad/miR-TFG) and scrambled microRNA (Ad/miR-Scr). Cells were observed under the fluorescent microscopy. Almost all the cells were transduced by the recombinant adenoviruses. (b) RT-PCR analysis showed that TFG was markedly down-regulated in Ad/miR-TFG-treated cells compared to control adenovirus (Ad/miR-Scr)-treated cells.

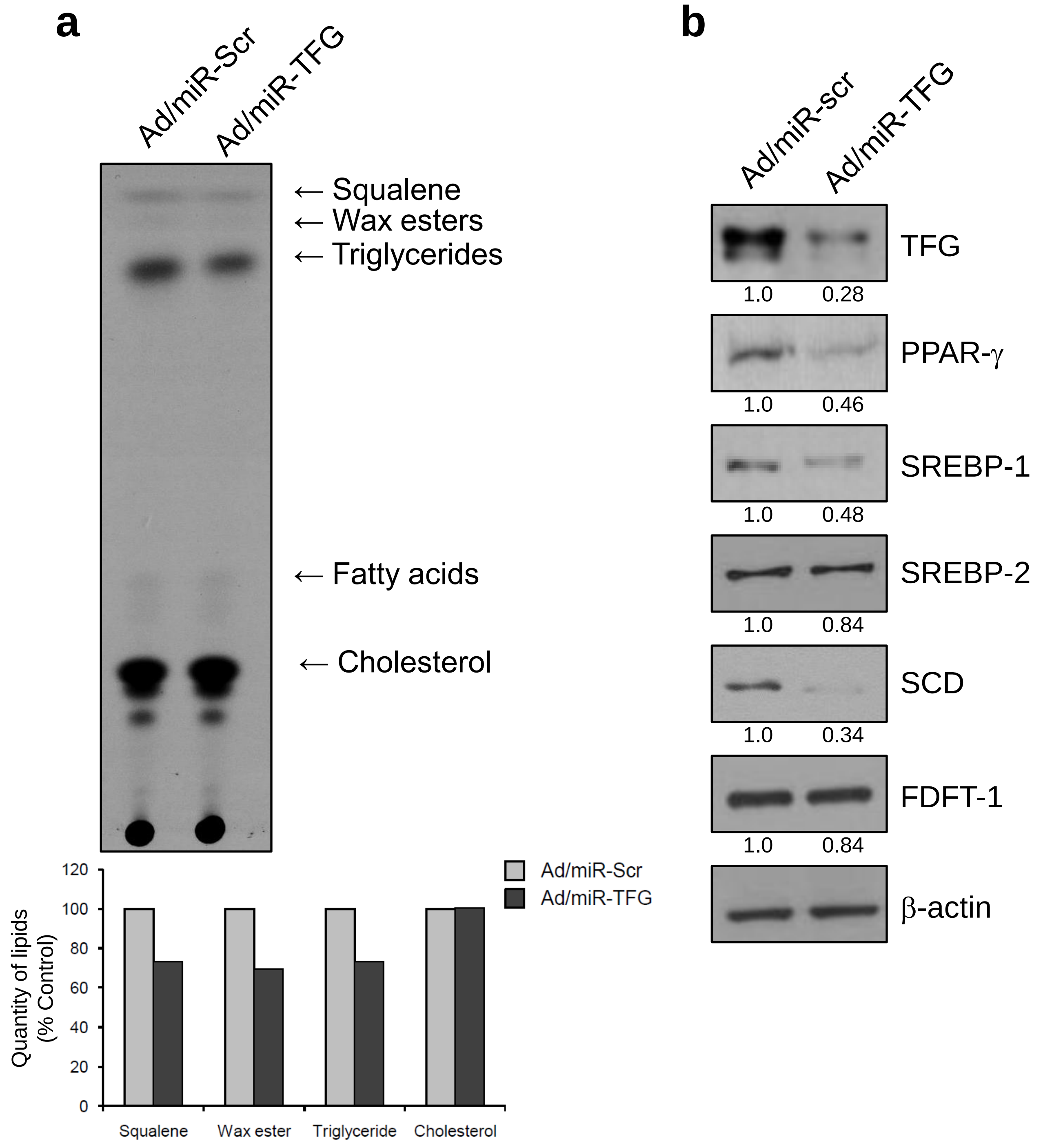

Supplementary Figure S4. (a) Primary cultured human sebocytes were transduced with the recombinant adenovirus expressing microRNA targeting TFG (Ad/miR-TFG). Scrambled microRNA (Ad/miR-Scr) was used as a negative control. Lipid production was analyzed by TLC. Down-regulation of TFG decreased the production of lipids including squalene and triglyceride. Quantification of lipids was carried out using ImageJ program. Data are represented as a percentage of the control. (b) Down-regulation of TFG decreased the protein levels for lipogenic regulators such as SREBP-1 and SCD. The protein levels were normalized to β-actin and represented as fold induction.

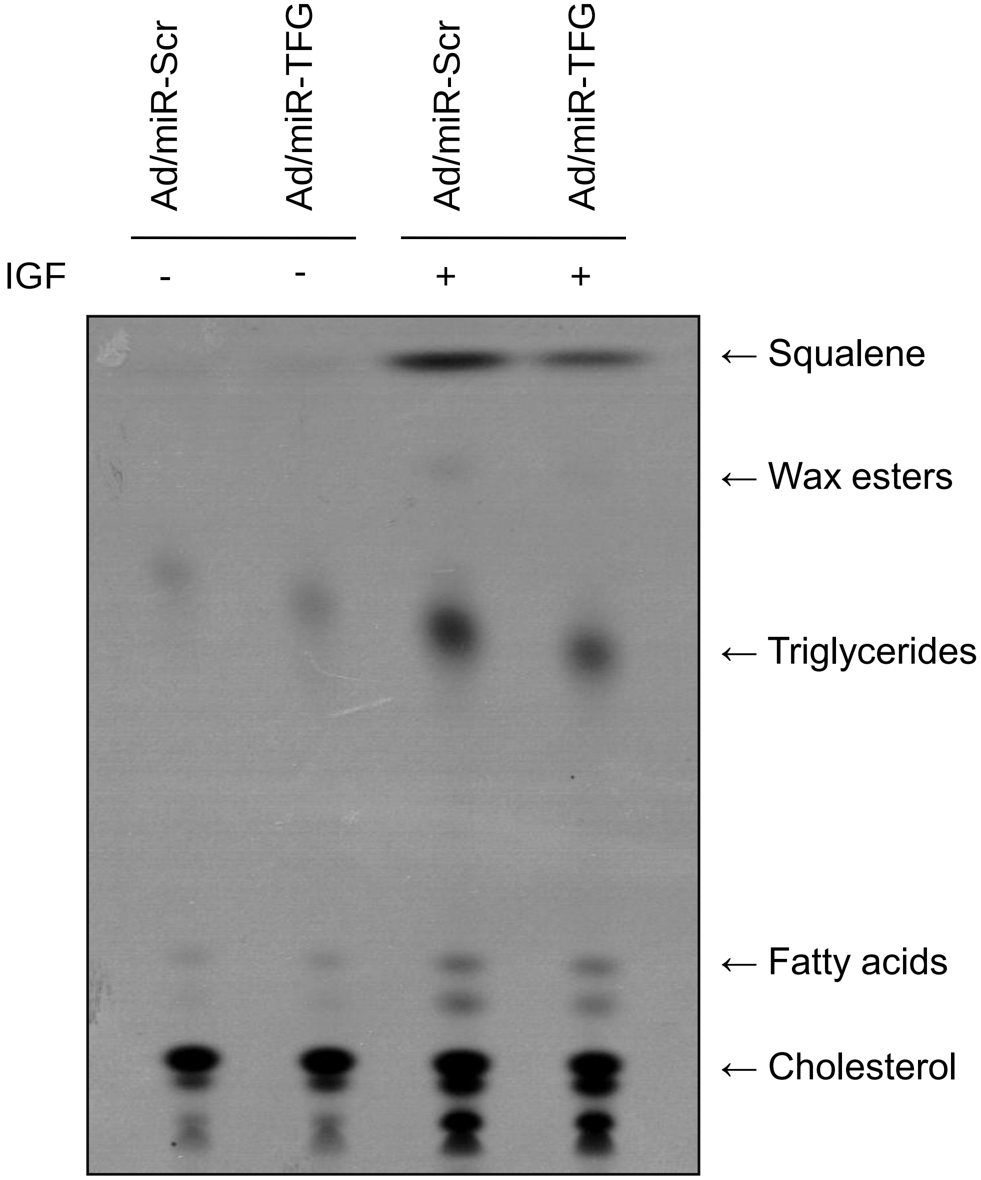

Supplementary Figure S5. SV-sebocytes were transduced with the recombinant adenovirus expressing microRNA targeting TFG (Ad/miR-TFG) or scrambled microRNA (Ad/miR-Scr). Cells were replenished with fresh medium without FBS and rhEGF. Cells were treated with IGF-1 (50 ng/ml) for 2 d. Intracellular lipids were analyzed by TLC. In Ad/miR-TFG-treated cells, IGF-1-induced lipogenesis was slightly inhibited compared to Ad/miR-Scr-treated cells.

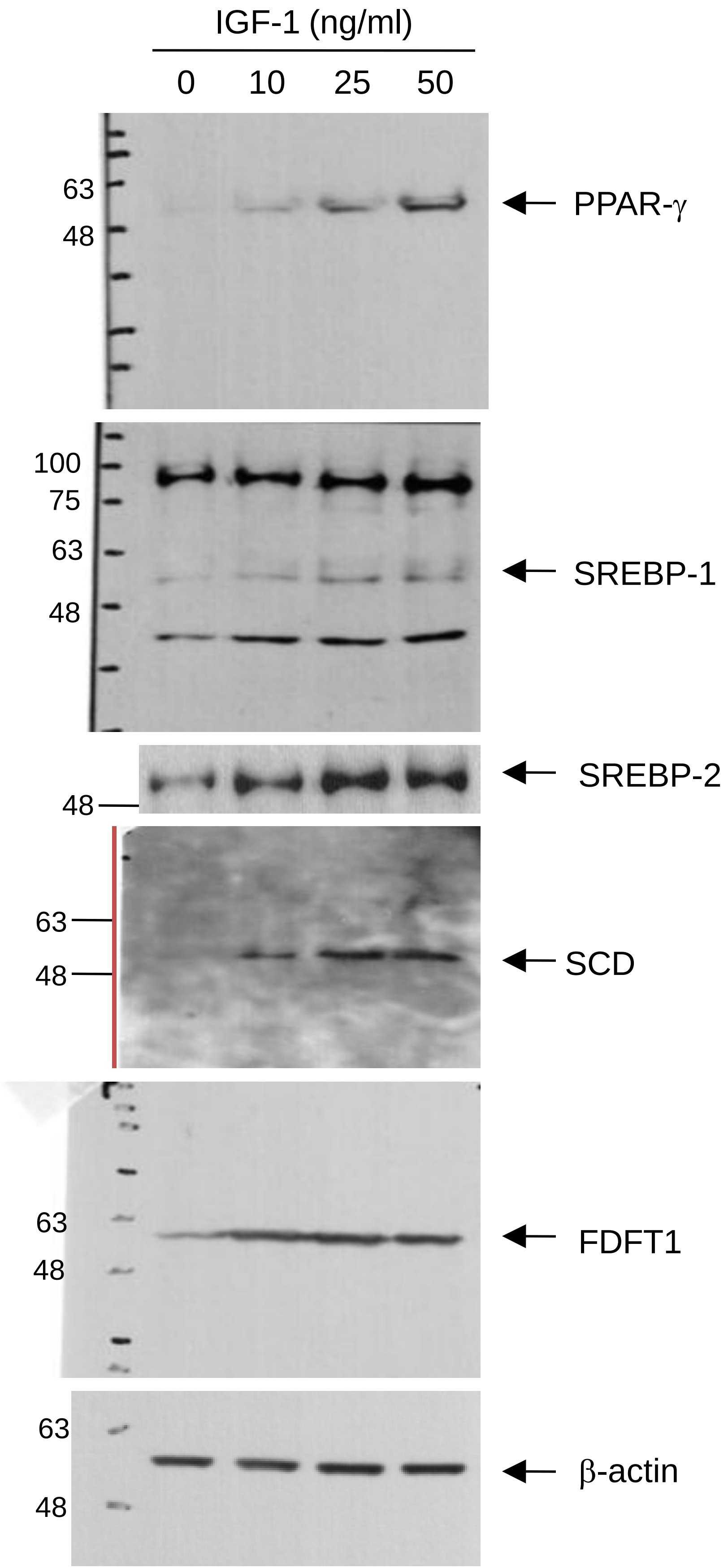

Supplementary Figure S6. Uncropped data for Figure 1d.

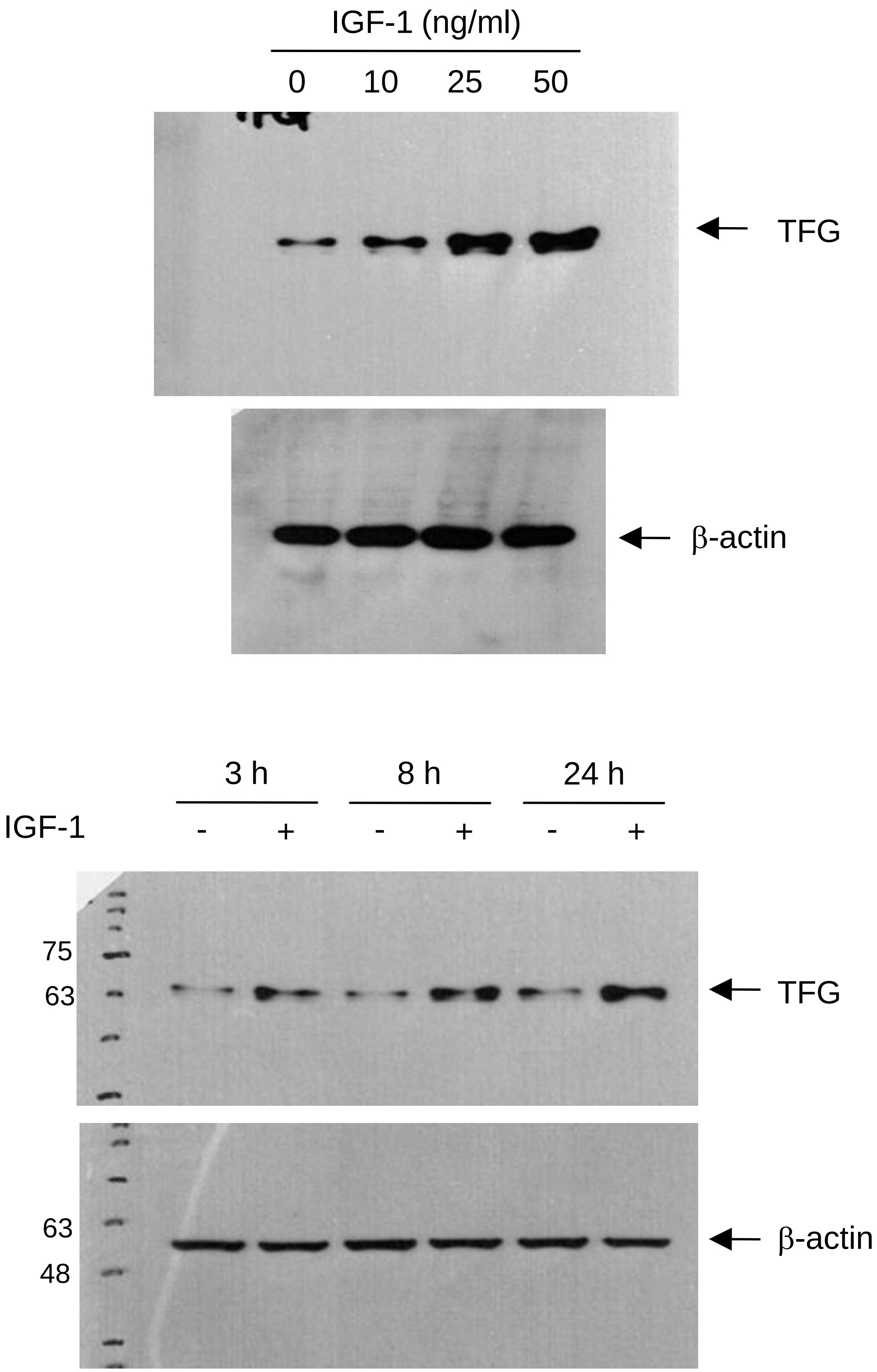

Supplementary Figure S7. Uncropped data for Figure 2a.

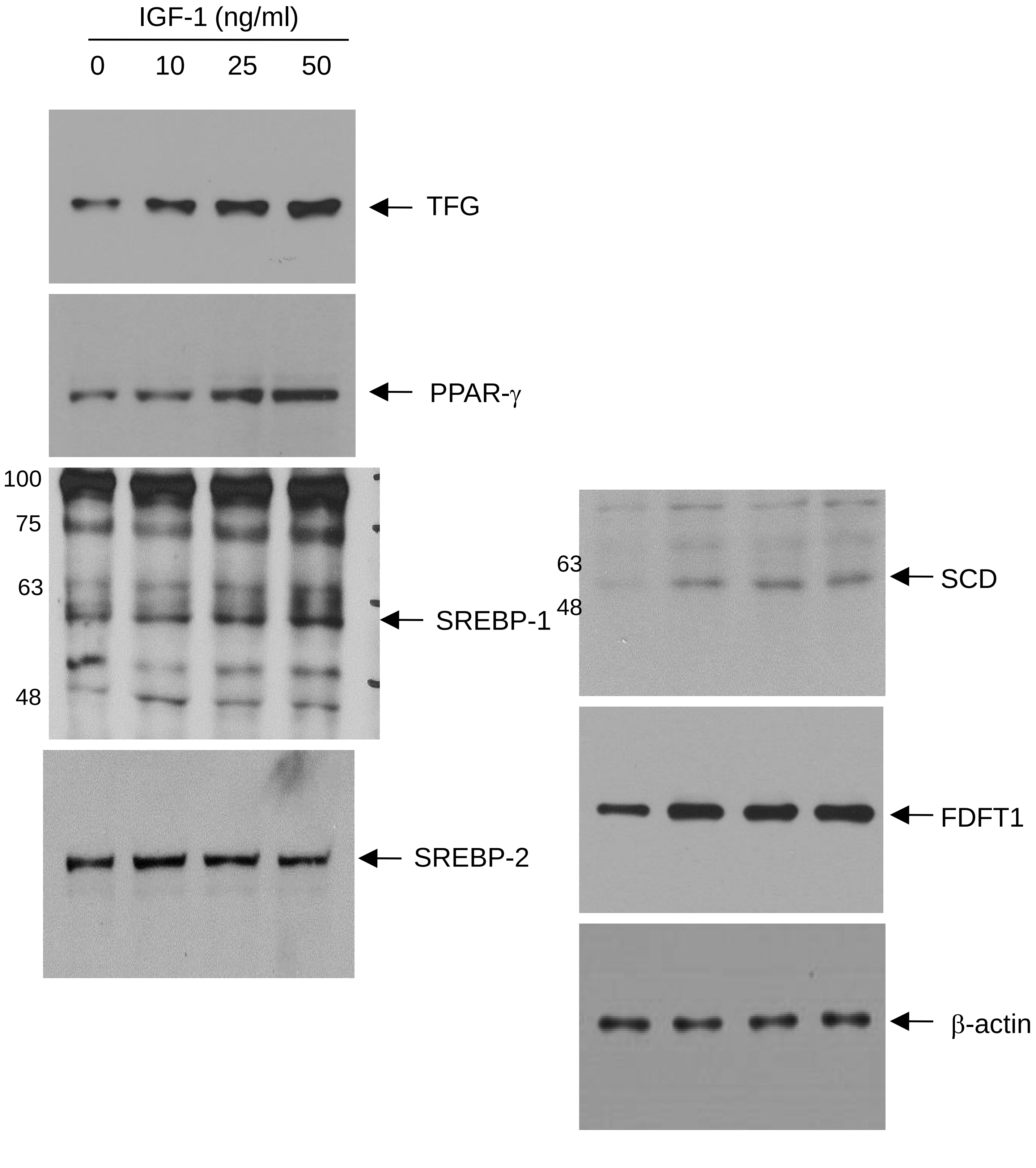

Supplementary Figure S8. Uncropped data for Figure 2b.

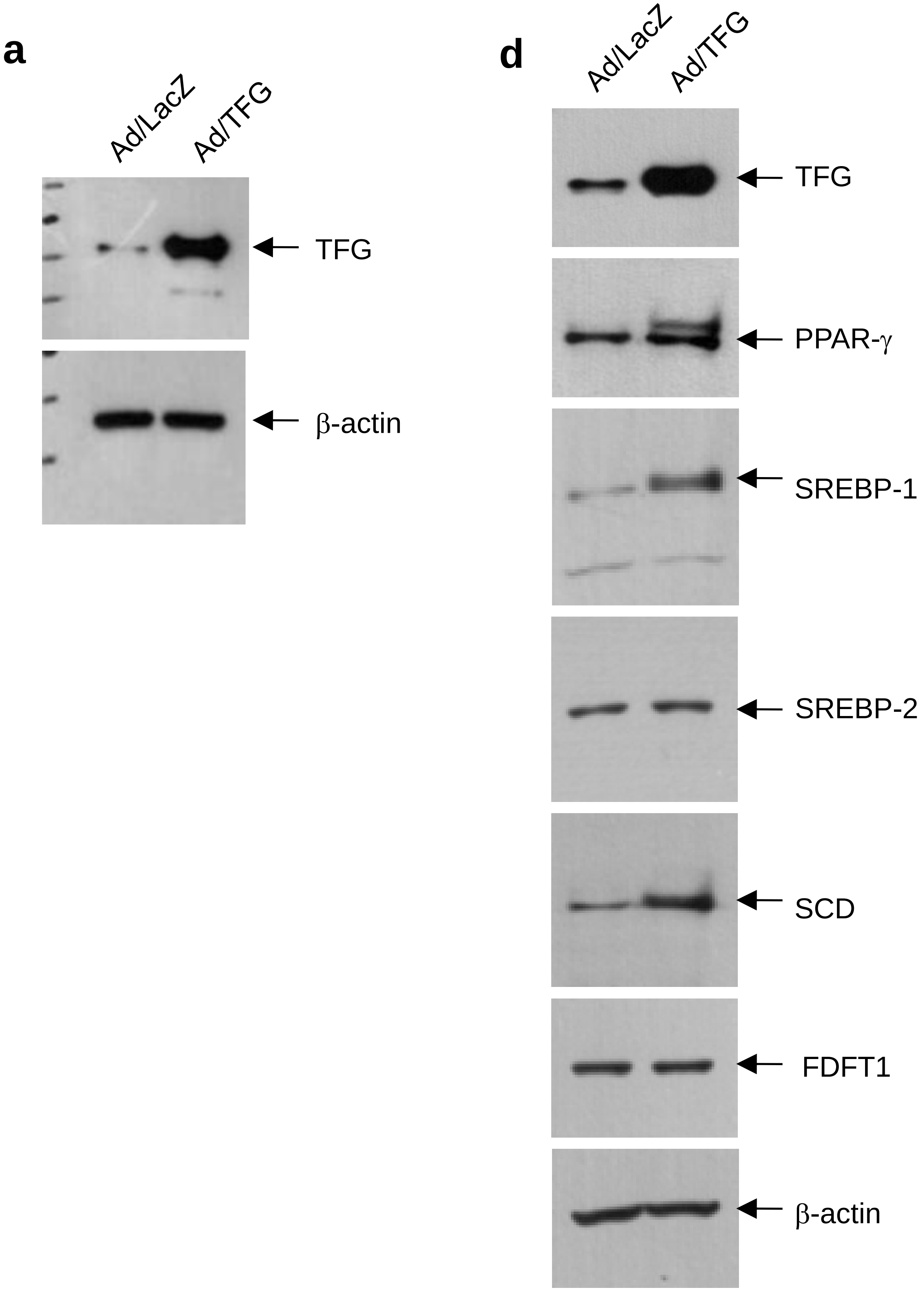

Supplementary Figure S9. Uncropped data for Figure 3a and Figure 3d.

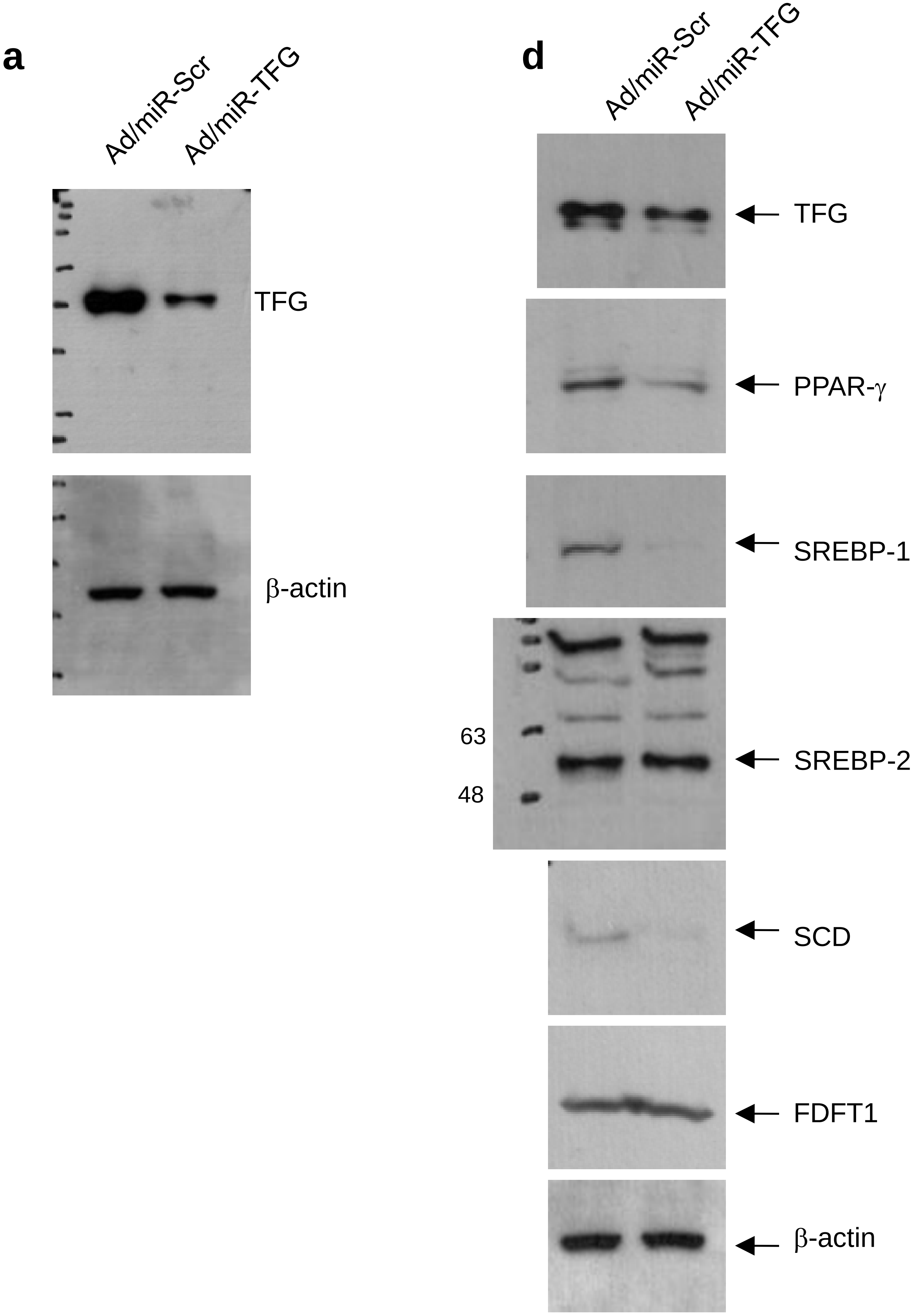

Supplementary Figure S10. Uncropped data for Figure 4a and Figure 4d.
